# Challenges and Opportunities for Exploring Patient-Level Data

**DOI:** 10.1155/2015/150435

**Published:** 2015-10-04

**Authors:** Pedro Lopes, Luis Bastião Silva, José Luis Oliveira

**Affiliations:** Department of Electronics, Telecommunications and Informatics (DETI), Institute of Electronics and Informatics Engineering of Aveiro (IEETA), University of Aveiro, 3810 193 Aveiro, Portugal

## Abstract

The proper exploration of patient-level data will pave the way towards personalised medicine. To better assess the state of the art in this field we identify the challenges and uncover the opportunities for the exploration of patient-level data through the review of well-known initiatives and projects focusing on the exploration of patient-level data. These cover a broad array of topics, from genomics to patient registries up to rare diseases research, among others. For each, we identified basic goals, involved partners, defined strategies and key technological and scientific outcomes, establishing the foundation for our analysis framework with four pillars: control, sustainability, technology, and science. Substantial research outcomes have been produced towards the exploration of patient-level data. The potential behind these data will be essential to realise the personalised medicine premise in upcoming years. Hence, relevant stakeholders continually push forward new developments in this domain, bringing novel opportunities that are ripe for exploration. Despite last decade's translational research advances, personalised medicine is still far from being a reality. Patients' data underlying potential goes beyond daily clinical practice. There are miscellaneous challenges and opportunities open for the exploration of these data by academia and business stakeholders.

## 1. Introduction

The widespread collection of patient-level data represents a critical step towards the realization of personalised medicine [1, 2]. These data stem from primary care centres, hospital information systems, clinical trials' cohorts, and administrative platforms. Moreover, they withhold a huge potential that goes beyond daily clinical care [3, 4].

Yet, along with the miscellaneous opportunities to explore patient-level data, this unparalleled growth of patients' digital metadata brings several challenges [5, 6]. Data size, lack of open access, heterogeneity, or the uses of primitive technologies are some of the issues researchers face [7]. In contrast, exploring the potential behind these data will lead to the discovery of new knowledge, essential to improve the current clinical narrative [8, 9].

Although patient-level data from public institutions, such as hospitals or regional/national administration centres, should be easier to access, it is generally locked under primitive technological implementations. This results in closed data silos that hinder scientific and technological evolution. Several large-scale projects already try to commoditize access to these data, whether through policies or through technical standards for data exchanges [10].

Pharmaceutical companies are also responsible for a big chunk of patient-level data [11]. Clinical trials' cohorts generate comprehensive patient datasets whose value for personalised medicine research is immeasurable [12, 13]. Despite this, most of pharmaceutical data are private [14].

It is important to distinguish between private companies' data, which is the basis for internal research and development for new drugs and treatments, from public research datasets, fundamental to advance general scientific research. Although pharmaceutical companies are entitled to keep their results private, policies should be put in place to foster the sharing of clinically relevant results into the public domain.

Dealing with this heterogeneous mixture of private and public patient-level data, tools, standards, and projects is in itself a complex research and development challenge [15]. Ultimately, the entropy in this ecosystem is delaying what should be a swift evolution. Hence, we need to evaluate past and on-going initiatives to better assess and plan the personalised medicine research and development roadmap for the upcoming years [16].

For this matter we established an evaluation framework to analyse the outcomes of existing initiatives, identifying current challenges and uncovering new opportunities. This framework is based on four key pillars: control, sustainability, technology, and science. We assess several components in each of these areas, generating a rather comprehensive study:

- the control section focuses on data ownership and access;
- the sustainability topics cover the long-term perspectives for each asset;
- on technology we assess the technical outcomes for each project, where existing;
- at the science level we identify the projects' research areas and their key scientific outcomes.

We present this comprehensive review targeting three key objectives. These were to (1) identify the best initiatives dealing with patient-level data, (2) inspect and study their different features, and (3) evaluate tackled challenges and open opportunities. Furthermore, we shed some light on the current status of public investment into research, where the lack of strict evaluation guidelines brings too much liberty to funded project partners. This research work brings true added value to multiple fields in the scientific domain; from the performance analysis of hospital care [17, 18] to the on-going exploration of pharmaceutical trials data [19], among others [20].

## 2. Materials and Methods

### 2.1. Design

This review covers past and on-going large-scale projects. Selected projects' evaluation is based on an assessment framework with four key components: control, sustainability, technology, and science. This design allows us to better understand the projects' outcomes distribution as well as defining an initial categorization for each project. We chose topics for matching criteria in each area based on mappings with existing ontologies, namely, Simple Knowledge Organization System (SKOS) [21] and EMBRACE Data and Methods (EDAM) [22].

At the control level we assess several topics, detailed next.

- *Data ownership*: who owns the project data and who decides whether to make data available or not? Available options are* community*,* partner*, or* project*.
- *Data access*: is there open access to the project's data or is it closed to project partners? Available options are* partners only*,* private*, or* public*.
- *Data storage*: are data stored in partners' private repositories or publicly shared with the involved community? Available options are* partners only*,* private*, or* public*.
- *Patient involvement*: are patients engaged in data ownership; that is, can patients control who can use their personal data in the project's systems? Available options are* no* or* yes*.
- *Security, privacy, and auditing*: how are security, privacy, and auditing issues dealt with within the project? Available options are* external*,* none*, or* project*.

In this review we also assess the selected projects' sustainability, covering the following areas:

- *Business model*: what is the business model behind the data owners? This has implications on what happens beyond each project's scope. Available options are* academia*,* business*, or* undefined*.
- *Data maintenance*: associated with the project's partners' business model, we have to assess what will happen with the collected data when the project finishes. Available options are composed of* stored*,* unpublished*, or* undefined*.

At the technology level we identified the technological outcomes from the studied projects, where available.

- *Technological outcomes*: are there (or will there be) any relevant technical outcomes from the project? Available options are* yes*,* only scientific*,* too soon to know*, or* undefined*.
- *Technology*: what are the main technological outcomes of each project? This includes* database*,* framework*,* infrastructure*,* library*,* standards*,* virtual machine*,* web services*, or* undefined*.

At last, we inspected the key scientific outcomes for each project, evaluating their areas of impact.

- *Field of research*: it is the fields of research with results that will have direct application to improve patient-level data exploration. These include* EHR*,* epigenomics*,* genomics*,* metabolomics*,* pharmacogenomics*,* phenomics*,* proteomics*,* transcriptomics*, and* other*.
- *Area of interest*: similarly to the field of research, we identified the technological areas of scientific interest that were studied in the project. Available options are* analytics*,* annotation*,* data integration*,* data visualization*,* ontology*,* semantic analysis*,* text-mining*, and* other*.

### 2.2. Inclusion and Exclusion Criteria

We searched for large-scale international projects in literature and general listings. From there, the inclusion criteria for this review were as follows:

- is on-going or finished after January 1st, 2011;
- is sponsored mainly by the NIH, IMI, or the European Commission;
- includes partners from both academia and the business sector;
- must focus on rare diseases, pharmacy or have direct patient involvement;
- must have public published results.

For all identified projects, we reviewed titles, funding information, references, and available publications to better assess if the projects appeared to meet all inclusion criteria. If insufficient information was available to make a confident decision, we contacted key project partners to disclose further details.

## 3. Results

This review provides an overview of the different attempts at improving the exploration of patient-level data. This section details the projects' evaluation according to our framework, including a tabular and visual comparison of their distinct features. From this evaluation we identify the main challenges and opportunities for future research endeavours.

### 3.1. Projects

Our initial dataset was extracted from the online project databases of three major funding agencies: USA's National Institutes of Health (NIH), European Commission (EC), and the Innovative Medicines Initiative (IMI) [23–25]. After a comprehensive filtering and selection process, 16 projects met our inclusion criteria (Table 1).

On a first glance we can quickly assess that the selected projects' domains and goals are heterogeneous, with the access or use of patient-level data being one of the few common threads. There is also an obvious bias towards European projects, as the European Commission continues to be a strong proponent of research, namely, on the life sciences and medical areas.

### 3.2. Feature Comparison

In this section we explore the projects' evaluation results according to the several pillars of our evaluation framework.

#### 3.2.1. Control

**Figure 1:**
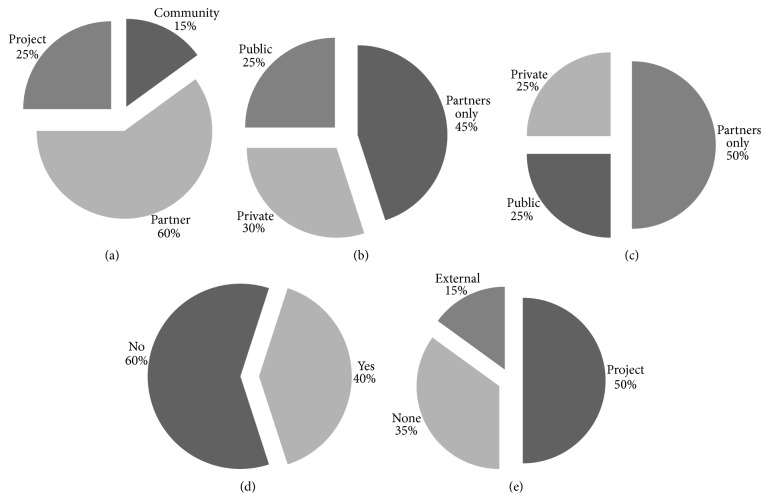
Data control evaluation breakdown charts. Charts summarizing evaluation results for the control section of the proposed evaluation framework. (a) Data ownership; (b) data access; (c) data storage; (d) patient involvement; (e) security, privacy, and auditing.

From Figure 1, highlighting the control pillar, we can conclude that there is real diversity in the projects being assessed regarding who controls the data. The notable exception concerns the patient involvement (Figure 1(d)). Although patients play a fundamental role in the research workflow, patients and patient advocacy groups are seldom considered as partners. As the other charts in Figure 1 show, data are equally distributed, owned, and stored by partners, the project, and the public domain. However, if we make a more basic categorization between open (public or community) and private (project or partner), the division is steeper.

#### 3.2.2. Sustainability

**Figure 2:**
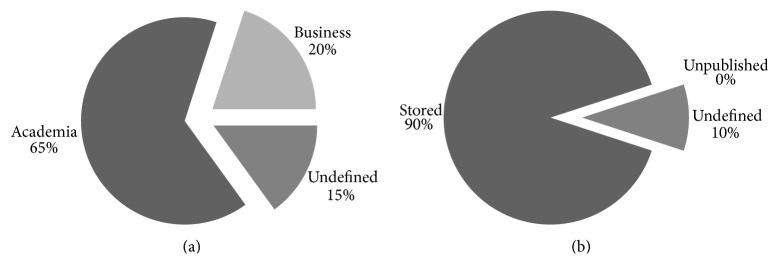
Data sustainability evaluation breakdown charts. These two charts feature the tracked sustainability topics in the proposed evaluation framework. (a) Business model and (b) data maintenance.

Our sustainability review entails better prospects for future data exploration. As Figure 2(b) highlights, the majority of projects already do or plan on doing active data maintenance. This implies that data collected within the project's scope will be stored for future use. Even if the access is limited, keeping these data alive opens good prospects for future endeavours. About half the evaluated projects will continue to provide their results to academia and some will focus on creating a business to sustain their research work once the project finishes (Figure 2(a)).

#### 3.2.3. Technology

**Figure 3:**
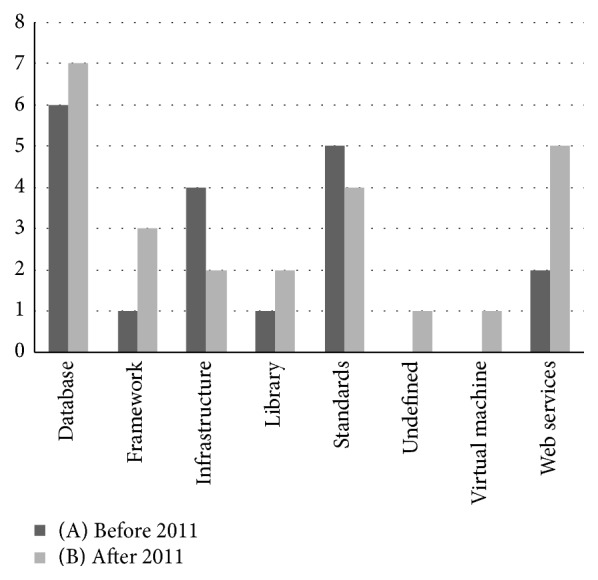
Technology outcomes' evaluation evolution breakdown chart. This chart features the key technological outcomes across the various projects, as assessed according to the proposed evaluation framework. To better understand the results' evolution over time, project evaluation results are divided between projects started before the year 2011 (A) and after the year 2011 (B).

At the technological level, all evaluated projects already produced public results. As expected from the heterogeneous project goals, there is an assorted amount of technical outcomes.  Figure 3 highlights the current trend, where services and databases are the focus of produced work. Next, infrastructure development is also a key area in selected projects, although they were more relevant for projects started before 2011 (Figure 3(A)). These particular results are of particular relevance for our review. We can infer that there is already proper effort put towards creating infrastructures for research. Hence, we should move our focus to the better exploration of existing resources, namely, with the creation of additional frameworks, standards, and services.

#### 3.2.4. Science

**Figure 4:**
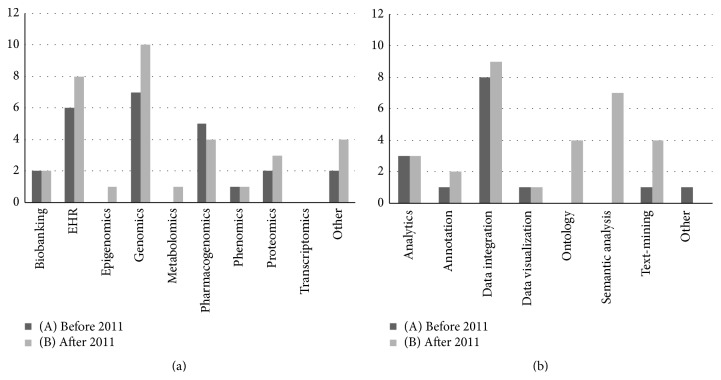
Science outcomes' evaluation evolution breakdown charts. Charts summarizing the various scientific research topics covered across the various projects assessed with the proposed evaluation framework. (a) Field of research; (b) area of interest. To better understand the results' evolution over time, project evaluation results are divided between projects started before the year 2011 (A) and after the year 2011 (B).

As shown in Figure 4, we find greatest variety of project features at the scientific level.  Figure 4(a) chart presents the various fields of research for projects started before 2011 (Figure 4(a)(A)) and after 2011 (Figure 4(a)(B)). In these, genomics is evidently important. Although the results are biased due to the selected projects' domain, there is a clear influence of genomics, pharmacogenomics, and biobanking at the patient-level domain (EHR). Nevertheless, as shown in Figure 4(a)(B), the miscellaneous omics research fields continue to be of interest and EHR interest is growing.

Figure 4(b) also validates the fundamental role of data integration in the various research fields. Nowadays, data integration expertise must be a vulgar commodity for life sciences and medical related research projects. More importantly, Figure 4(b)(B), for projects started after 2011, the differences in the fields of analytics, ontologies, text-mining, and semantic analysis are staggering. This reveals the growing significance of semantic web related technologies, as they complement analytics, ontologies, and text-mining features.

### 3.3. Challenges and Opportunities

With this evaluation we identified several challenges and opportunities. Challenges relate to data discovery, access, acquisition, and ownership. This brings several opportunities to deploy future solutions that fully explore the enormous amounts of patient-level data, using technological paradigms that projects are already supporting.

#### 3.3.1. Challenges

There is a clear dichotomy regarding data. Patient-level data is a very specific use case for exploration. While there are too many data scattered throughout multiple stakeholders, they are wildly difficult to obtain. The outcome of this is that, in the end, there is not enough data to generate statistically meaningful conclusions. Hence, we cannot discover or infer new knowledge because there is no access to a minimal amount of patient data. Along with distribution, data heterogeneity arises as a key challenge for exploring patient-level data. As shown in Figure 3, there are already several projects dealing with creating new and improving existing data standards for data sharing. However, these are far from being widely adopted throughout international stakeholders. Bioinformatics and pharmacogenomics projects also face these challenges [41]. Nevertheless, for these there are already adequate standards for data storage and exchange [42–45].

In the same vein, data translation also arises as a complex challenge for researchers. In addition to the obvious sense (translating data between multiple languages [46]), there is the data translation from a low-level free text data to structured information [47, 48]. Clinicians' reports traditionally include their notes in free text. These notes must be mapped to a shared domain, elevated from simple text to meaningful structured knowledge. Again, the growing relevance of text-mining and semantic web technologies, as highlighted before, is visible.

Data discovery, access, and acquisition are typical problems that can be solved by improving existing technologies and by focusing on their widespread adoption. Unlike these, data ownership is a much more complex issue. Dealing with data ownership involves tackling issues related with government's policies, stakeholders' interests, and projects' internal guidelines. In an ideal scenario, all patient-level data should be available for research purposes. This should be particularly enforced in publicly funded projects. Yet, this does not happen. As seen in Figure 1, projects' data ownership, storage, and access resort to closed solutions. In most cases, data are privately held, or at most, shared to project partners. Moreover, where data are shared publicly to researchers, access restrictions are in place.

#### 3.3.2. Opportunities

Great challenges leverage great opportunities. From our review, we believe there is room for improving how we explore patient-level data and how we can use it to further improve research and development towards personalised medicine. As Figure 4 highlights, on-going projects are already solving important technological challenges.

There is huge potential behind the combination of data available worldwide. Yet, we need to develop and disseminate new technologies that improve how relevant entities collect, store, and share patient-level data.

As data integration is already commonplace, to obtain real advances in this domain we must see worldwide patient-level data as a whole, and not as single detached data silos. Although we already have the technology to accomplish this, stakeholders must unite efforts to make this holistic view a reality.

At the technical level, opportunities arise that demand the creation of new software and new standards. Likewise, at a policy level, we must improve existing guidelines and policies to better cover data sharing and ownership and ethics issues.

New data management standards should promote better (and easier) ways to access and share data. This will promote knowledge discovery and enable the integration and interoperability among patient-level data silos throughout the world. Likewise, going from patient-level data to summary-level data, and vice-versa, should be a simple straightforward process with the latest text-mining and semantic web tools.

Ideally, new software will empower collaboration and sharing among patients and clinicians. These should promote ease of access to patient information and enhance the communication process among clinicians. Furthermore, new tools are required to enhance data ownership controls, facilitating how patients, clinicians, or researchers express who has access to relevant personal data. More importantly, a combination of policies and guidelines should be put in place to foster the active involvement of patients in clinical care.

Despite the great opportunity for creating new standards and software, these assets alone are not enough to change the current scenario. New politics and guidelines, stemming directly from key worldwide stakeholders, must be disseminated to all interested parties. Moreover, with adequate support from governmental agencies (regional, national, and international), projects and their internal partners will proactively work towards implementing these new guidelines.

## 4. Discussion

As this review reveals, there is room for change in the exploration of patient-level data. However, we must take in account that these results are biased and strict. This is an ever-expanding field with lots of partners, projects, and companies working in this subject.

While we tried to be comprehensive, this review has obvious limitations. Namely, identifying each project's features and technical/scientific outcomes was a complex task. Once the projects finish, little to no effort is put into maintaining an accurate dissemination summary and rarely the projects results are assessed a couple years after each project's conclusion.

### 4.1. The Growing Relevance of Genomics Data

The core focus of this review revolves around projects dealing with patient-level data stemming from electronic patient records. However, as shown in Figure 4(a), the quantity and quality of projects interacting with patient databases focused on genomics data are growing [49]. Furthermore, next generation sequencing technologies streamline the generation of huge patient datasets [50].

In a sense, patient sequencing data are patient-level data. Projects, such as 1000 Genomes [51] or Genome of the Netherlands [52], are trying to sequence large numbers of individuals to better understand existing genotype-phenotype relationships and uncover new ones.

In the long term, these data will be included in clinical patient registries. They may even be part of the electronic patient record. At this stage, clinicians will require new tools to adequately exploit the true value behind these data. In summary, this is a whole new field of exploration for personalised medicine and patient-level data research that cannot be ignored [53].

### 4.2. Implications for Future Research

As detailed in previous sections, the various opportunities highlight the room for improvement in this domain. Assessing the projects' timing evolution we identify that the focus on sharing, dissemination, and patient control is of growing relevance in the field.

The creation of new technical standards and data sharing policies will be fundamental for future research. Moreover, these topics are emerging in current project calls. Thus, they are becoming a stepping-stone for future research and infrastructure initiatives.

Despite the scale of on-going projects, they will not cover every possible topic. Technological developments in analytics tools, text-mining, ontologies, semantic web, data visualisation, integration, and interoperability, originating from distinct areas, must be brought to patient-level exploration.

The semantic web arises as a ground breaking paradigm to foster the intelligent integration of structured information. Sustained by state-of-the-art standards such as RDF, OWL, SPARQL, and LinkedData, semantic web promotes better strategies to express, infer, and make knowledge interoperable.

Latest advances in the area cover the research and development of new algorithms to further improve how we collect data, transform data into meaningful knowledge assertions, and publish connected knowledge. To further improve this, we must rely on the latest text-mining technologies. Elevating clinical text data to abstract knowledge or mapping the best matching ontologies to patient datasets require advanced text-mining solutions.

The combination of these strategies, semantic web, text-mining, and ontologies will pave the way towards interoperable scientific knowledge. These technologies will foster data integration and interoperability, enabling an effortless connection between heterogeneous distributed knowledge, obtained from patient-level data. Hence, the foundation of translational research, where multiple technical research areas collide, will be even more meaningful in the future.

### 4.3. Impact

Although this review had the main goal of covering the scientific results, we cannot ignore additional fundamental questions surrounding large-scale projects.

Hence, we must discuss the privacy policies applied to research-oriented datasets, the creation of businesses sustained by public funding, or the lack of publicly visible project evaluation outcomes.

The general community perceives that there is a huge amount of public funds being poured into research projects in all areas. Still, the outcomes of these projects are not as public as desired. There is an underlying sense of fulfilment in investing on research, especially in fields related with life sciences, such as rare diseases treatments, pharmaceutical research, or any other relevant omics field: IMI, EC, and NIH are funding science.

Figure 1(b) highlights that only a quarter of studied projects expect to provide their data publicly to the general research audience. Data access restrictions are too common on research. Large investments, with public funds, are being applied to clinical drug trials, patient registries development, and next generation sequencing technologies. Yet, the majority of research outcomes will not be made available to the public. And, despite pharmaceutical companies financial involvement in IMI projects, the expected profit outcome from these projects will definitely surpass invested money. Patient-level data, obtained with public research funds, which have the potential of being fundamental to create new knowledge, are not available to the research community as they are closed behind complex privacy policies and never-ending access restrictions.

Likewise, Figure 2 charts show that there are several projects whose future sustainability will rely on implementing a profit-oriented business model. Hence, we must ask, again, how can public funds, applied to research projects, be used to create self-sustainable companies? These companies will sell products, software or data, created with research funds stemming from public investment.

At last, there is a great difficulty in finding projects details and their respective evaluation results. It is as if the IMI, EC, and NIH projects lists are difficult to access and lack essential project details on purpose. The general audience cannot find out how projects are evaluated, their assessment results and, more importantly, their visible outcomes. Despite having concluded that most project results are private, the projects' evaluation should be public. Furthermore, it should be supported by a clear long-term plan that assessed the proper use of public funds to actually advance research. Finished projects should be evaluated in multiple timespans, not just when the deadline is reached. Evaluating projects 2, 5, or 10 years after their finish date would improve the understanding of how successful was the large sum of invested money.

The reality is that IMI, EC, and NIH are funding projects that have the liberty to create for-profit businesses and, more importantly, the liberty to apply public funds to the most diverse research tasks, whether they are directly related to the expected project results.

## 5. Conclusions

This review provides an overview of different initiatives that try to properly explore patient data. We limited our study to research and development projects in the recent past. We established base criteria to evaluate on-going initiatives. This resulted in the identification of several opportunities for future developments, namely, (1) bringing distributed data together by putting more advanced sharing and integration at clinicians' fingertips; (2) focus on text-mining and semantic web technologies to create real knowledge from distributed and heterogeneous data; and (3) pressuring stakeholders for stricter project evaluations that will foster a quicker evolution pace. The lack of well-established and widely adopted solutions covering these areas represents a major roadblock for the adequate exploration of patient-level data. However, if future projects consistently adopt these overarching goals, personalised medicine will be one step closer.

More importantly, in addition to the research-specific evaluation outcomes, we must highlight the strange patterns behind large-scale project funding. Although IMI, NIH, and EC provide intensive financial support for research, what we witness is that the money is being used to create for-profit businesses and closed research datasets. Furthermore, funding agencies lack clear evaluation frameworks that properly assess the success of public investment into large-scale research.

## Figures and Tables

**Table 1:** List of evaluated projects.

Project	Start	End	URL	Description
BBMRI	2008	2011	http://bbmri.eu/	BBMRI connects researchers, biobankers, patient advocacy groups, and pharmaceutical research companies to foster a quicker discovery of new treatments [26]. Their strategy is based on the enrichment and harmonization of biobanks.

BioMedBridges	2012	2015	http://www.biomedbridges.eu/	BioMedBridges' goal is to launch a shared e-infrastructure for biological and biomedical data.

BioSHaRe-EU	2010	2015	https://www.bioshare.eu/	BioSHaRe-EU partners are working to ensure the development of harmonized measures and standardized computing infrastructures.

BRIDGEtoData	2011	—	http://www.bridgetodata.org/	BRIDGEtoData aims to be an online reference platform describing population healthcare databases for use in epidemiology and health outcomes research.

DDMoRe	2011	2016	http://www.ddmore.eu/	The Drug Disease Model Resources (DDMoRe) project aims to establish a universal standard framework for modelling drugs and diseases [27, 28].

EHR4CR	2011	2014	http://www.ehr4cr.eu/	EHR4CR partners built, validated, and deployed a Europe-wide innovative technological platform to reuse EHRs data for clinical research purposes [29].

ELIXIR	2010	2018	http://www.elixir-europe.org/	ELIXIR project's goal is to coordinate the collection, quality control, and archiving of large amounts of biological data [30].

EMIF	2012	2018	http://www.emif.eu/	EMIF's goal involves the creation of an innovative and connected patient registry catalogue that will enable researchers and pharmaceutical companies to search for patient-level data based on the databases' digital fingerprints [31].

ESGI	2011	2015	http://www.esgi-infrastructure.eu/	ESGI's goal is to integrate and standardise current and emerging technologies, providing access to infrastructures so that a broad group of European researchers can use the new technologies.

eTRIKS	2012	2017	http://www.etriks.org/	eTRIKS' objective is to address knowledge management gaps by building a sustainable translational research informatics/knowledge management platform and to provide additional sustainable services.

EU-ADR	2008	2012	https://bioinformatics.ua.pt/euadr/	EU-ADR project aimed developing a unique computerized system to detect adverse drug reactions (ADRs), supplementing spontaneous reporting systems [32].

EURenOmics	2012	2018	http://www.eurenomics.eu/	EURenOmics work is based on rare kidney diseases, where the project seeks to establish more accurate diagnoses strategies and improve clinical care.

Euro-BioImaging	2010	2014	http://www.eurobioimaging.eu/	Euro-BioImaging's main work covered the improvement of existing research infrastructures on a large scale.

GEN2PHEN	2008	2013	http://gen2phen.org/	GEN2PHEN was created to unify human and model organism genetic variation databases towards increasingly holistic views into Genotype-to-Phenotype (G2P) data and to link this system into other biomedical knowledge sources via genome browser functionality [33].

NeurOmics	2012	2018	http://rd-neuromics.eu/	NeurOmics' research objectives feature the study of neurodegenerative and neuromuscular diseases in an attempt to explore Omics technologies to improve diagnosis, treatments, and general patient care.

OMOP	2008	2013	http://omop.org/	OMOP's goal was to design experiments testing a variety of analytical methodologies in a range of data types to look for drug impacts, going towards a complete database analysis standard [34].

Oncotrack	2011	2016	http://www.oncotrack.eu/	Oncotrack deploys several methods for systematic next generation oncology biomarker development [35, 36].

OpenPHACTS	2011	2014	http://www.openphacts.org/	OpenPHACTS works with the integration of a relevant and continuously expanding subset of distributed heterogeneous data sources into one “virtual resource,” via the creation of a semantic interoperability layer [37].

RD-Connect	2012	2018	http://rd-connect.eu/	RD-Connect will launch an integrated platform connecting databases, registries, biobanks, and clinical bioinformatics for rare diseases research [38].

Sentinel	2008	—	http://www.fda.gov/Safety/FDAsSentinelInitiative/default.htm	Sentinel is a USA-based electronic system that will transform FDA's ability to track the safety of drugs, biologics, and medical devices [39, 40]. This initiative aims to develop and implement a proactive system that will complement existing systems that the FDA has in place to track reports of adverse events linked to the use of its regulated products.

## References

[1] Miles A., Loughlin M., Polychronis A. (2008). Evidence-based healthcare, clinical knowledge and the rise of personalised medicine. *Journal of Evaluation in Clinical Practice*.

[2] Hamburg M. A., Collins F. S. (2010). The path to personalized medicine. *The New England Journal of Medicine*.

[3] Harvey A., Brand A., Holgate S. T. (2012). The future of technologies for personalised medicine. *New Biotechnology*.

[4] Coorevits P., Sundgren M., Klein G. O. (2013). Electronic health records: new opportunities for clinical research. *Journal of Internal Medicine*.

[5] Lyman G. H., Kuderer N. M. (2005). The strengths and limitations of meta-analyses based on aggregate data. *BMC Medical Research Methodology*.

[6] Coloma P. M., Schuemie M. J., Trifirò G. (2011). Combining electronic healthcare databases in Europe to allow for large-scale drug safety monitoring: the EU-ADR Project. *Pharmacoepidemiology and Drug Safety*.

[7] Tudur Smith C., Williamson P. R., Marson A. G. (2005). Investigating heterogeneity in an individual patient data meta-analysis of time to event outcomes. *Statistics in Medicine*.

[8] Broeze K. A., Opmeer B. C., van der Veen F., Bossuyt P. M., Bhattacharya S., Mol B. W. J. (2010). Individual patient data meta-analysis: a promising approach for evidence synthesis in reproductive medicine. *Human Reproduction Update*.

[9] Xu H., Fu Z., Shah A. Extracting and integrating data from entire electronic health records for detecting colorectal cancer cases.

[10] Marceglia S., Fontelo P., Ackerman M. J. (2015). Transforming consumer health informatics: connecting CHI applications to the health-IT ecosystem. *Journal of the American Medical Informatics Association*.

[11] Wieseler B., Wolfram N., McGauran N. (2013). Completeness of reporting of patient-relevant clinical trial outcomes: comparison of unpublished clinical study reports with publicly available data. *PLoS Medicine*.

[12] Nisen P., Rockhold F. (2013). Access to patient-level data from GlaxoSmithKline clinical trials. *The New England Journal of Medicine*.

[13] Johnson D. E. (2013). Fusion of nonclinical and clinical data to predict human drug safety. *Expert Review of Clinical Pharmacology*.

[14] Sampat B. N., Lichtenberg F. R. (2011). What are the respective roles of the public and private sectors in pharmaceutical innovation?. *Health Affairs*.

[15] Daniel C., Albuisson E., Dart T., Avillach P., Cuggia M., Guo Y., Venot A., Burgun A., Quantin C. (2014). Translational bioinformatics and clinical research informatics. *Medical Informatics, e-Health*.

[16] Abernethy A. P., Ahmad A., Zafar S. Y., Wheeler J. L., Reese J. B., Lyerly H. K. (2010). Electronic patient-reported data capture as a foundation of rapid learning cancer care. *Medical Care*.

[17] Sutton A. J., Kendrick D., Coupland C. A. C. (2008). Meta-analysis of individual- and aggregate-level data. *Statistics in Medicine*.

[18] Olsen K. R., Street A. (2008). The analysis of efficiency among a small number of organisations: how inferences can be improved by exploiting patient-level data. *Health Economics*.

[19] Jain K. K. (2005). Personalised medicine for cancer: from drug development into clinical practice. *Expert Opinion on Pharmacotherapy*.

[20] Cuellar A. E., Gertler P. J. (2006). Strategic integration of hospitals and physicians. *Journal of Health Economics*.

[21] Miles A., Matthews B., Wilson M., Brickley D. SKOS core: simple knowledge organisation for the web.

[22] Ison J., Kalaš M., Jonassen I. (2013). EDAM: an ontology of bioinformatics operations, types of data and identifiers, topics and formats. *Bioinformatics*.

[23] Innovative Medicines Initiative I http://www.imi.europa.eu/content/ongoing-projects.

[24] Publications Office of the European Union (OP) CORDIS Projects and Results. http://cordis.europa.eu/projects/home_en.html.

[25] National Institutes of Health (NIH) NIH Awards. http://www.report.nih.gov/award/index.cfm.

[26] Wichmann H.-E., Kuhn K. A., Waldenberger M. (2011). Comprehensive catalog of European biobanks. *Nature Biotechnology*.

[27] Mentré F., Chenel M., Comets E. (2013). Current use and developments needed for optimal design in pharmacometrics: a study performed among DDMoRe's european federation of pharmaceutical industries and associations members. *CPT: Pharmacometrics & Systems Pharmacology*.

[28] Harnisch L., Matthews I., Chard J., Karlsson M. O. (2013). Drug and disease model resources: a consortium to create standards and tools to enhance model-based drug development. *CPT: Pharmacometrics & Systems Pharmacology*.

[29] El Fadly A., Rance B., Lucas N. (2011). Integrating clinical research with the Healthcare Enterprise: from the RE-USE project to the EHR4CR platform. *Journal of Biomedical Informatics*.

[30] Crosswell L. C., Thornton J. M. (2012). ELIXIR: a distributed infrastructure for European biological data. *Trends in Biotechnology*.

[31] Gottwald M. (2014). How can the innovative medicines initiative help to make medicines development more efficient?. *Re-Engineering Clinical Trials: Best Practices for Streamlining the Development Process*.

[32] Coloma P. M., Schuemie M. J., Trifirò G. (2011). Combining electronic healthcare databases in Europe to allow for large-scale drug safety monitoring: the EU-ADR Project. *Pharmacoepidemiology and Drug Safety*.

[33] Webb A. J., Thorisson G. A., Brookes A. J. (2011). An informatics project and online ‘Knowledge Centre’ supporting modern genotype-to-phenotype research. *Human Mutation*.

[34] Schuemie M. J., Gini R., Coloma P. M. (2013). Replication of the OMOP experiment in europe: evaluating methods for risk identification in electronic health record databases. *Drug Safety*.

[35] Elsner M. (2011). OncoTrack tests drugs in virtual people. *Nature Biotechnology*.

[36] Henderson D., Ogilvie L. A., Hoyle N., Keilholz U., Lange B., Lehrach H. (2014). Personalized medicine approaches for colon cancer driven by genomics and systems biology: oncoTrack. *Biotechnology Journal*.

[37] Harland L., ten Teije A., Völker J., Handschuh S. (2012). Open PHACTS: a semantic knowledge infrastructure for public and commercial drug discovery research. *Knowledge Engineering and Knowledge Management*.

[38] Thompson R., Johnston L., Taruscio D. (2014). RD-Connect: an integrated platform connecting databases, registries, biobanks and clinical bioinformatics for rare disease research. *Journal of General Internal Medicine*.

[39] Robb M. A., Racoosin J. A., Sherman R. E. (2012). The US food and drug administration's sentinel initiative: expanding the horizons of medical product safety. *Pharmacoepidemiology and Drug Safety*.

[40] Psaty B. M., Breckenridge A. M. (2014). Mini-sentinel and regulatory science—big data rendered fit and functional. *The New England Journal of Medicine*.

[41] Oliveira J. L., Lopes P., Nunes T. (2013). The EU-ADR Web Platform: delivering advanced pharmacovigilance tools. *Pharmacoepidemiology and Drug Safety*.

[42] Stevens R., Jupp S., Klein J., Schanstra J. Using semantic web technologies to manage complexity and change in biomedical data.

[43] Kohl M. (2011). Standards, databases, and modeling tools in systems biology. *Data Mining in Proteomics*.

[44] Jiménez R. C., Vizcaíno J. A. (2013). Proteomics data exchange and storage: the need for common standards and public repositories. *Mass Spectrometry Data Analysis in Proteomics*.

[45] Machado C. M., Rebholz-Schuhmann D., Freitas A. T., Couto F. M. (2015). The semantic web in translational medicine: current applications and future directions. *Briefings in Bioinformatics*.

[46] Pecina P., Dušek O., Goeuriot L. (2014). Adaptation of machine translation for multilingual information retrieval in the medical domain. *Artificial Intelligence in Medicine*.

[47] Rosenbloom S. T., Denny J. C., Xu H., Lorenzi N., Stead W. W., Johnson K. B. (2011). Data from clinical notes: a perspective on the tension between structure and flexible documentation. *Journal of the American Medical Informatics Association*.

[48] Rebholz-Schuhmann D., Oellrich A., Hoehndorf R. (2012). Text-mining solutions for biomedical research: enabling integrative biology. *Nature Reviews Genetics*.

[49] Schuster S. C. (2007). Next-generation sequencing transforms today's biology. *Nature Methods*.

[50] Mardis E. R. (2008). The impact of next-generation sequencing technology on genetics. *Trends in Genetics*.

[51] Via M., Gignoux C., Burchard E. G. (2010). The 1000 Genomes Project: new opportunities for research and social challenges. *Genome Medicine*.

[52] Boomsma D. I., Wijmenga C., Slagboom E. P. (2014). The Genome of the Netherlands: design, and project goals. *European Journal of Human Genetics*.

[53] Ware J. S., Roberts A. M., Cook S. A. (2012). Next generation sequencing for clinical diagnostics and personalised medicine: implications for the next generation cardiologist. *Heart*.

